# Environmental correlates of crimean-congo haemorrhagic fever incidence in Bulgaria

**DOI:** 10.1186/1471-2458-12-1116

**Published:** 2012-12-27

**Authors:** Fenicia M Vescio, Luca Busani, Lapo Mughini-Gras, Cristina Khoury, Luca Avellis, Evgenia Taseva, Giovanni Rezza, Iva Christova

**Affiliations:** 1 Division of Epidemiology, MIPI-Department of Infectious Diseases, National Public Health Institute, Rome, Italy; 2 Department of Veterinary Public Health and Food Safety, National Public Health Institute, Rome, Italy; 3 Division of Vector Borne Diseases, MIPI-Department of Infectious Diseases, National Public Health Institute, Rome, Italy; 4 National Reference Vector-Borne Infections and Leptospirosis Laboratory, National Center of Infectious and Parasitic Diseases, Sofia, Bulgaria

## Abstract

**Background:**

Crimean-Congo Haemorrhagic Fever (CCHF) is a zoonotic viral disease transmitted by ixodid tick bites, mainly of *Hyalomma* spp., or through contact with blood/tissues from infected people or animals. CCHF is endemic in the Balkan area, including Bulgaria, where it causes both sporadic cases and community outbreaks.

**Methods:**

We described trends of CCHF in Bulgaria between 1997 and 2009 and investigated the associations between CCHF incidence and a selection of environmental factors using a zero-inflated modelling approach.

**Results:**

A total of 159 CCHF cases (38 women and 121 men) were identified between 1997 and 2009. The incidence was 0.13 cases per 100,000 population/year with a fatality rate of 26%. An epidemic peak was detected close to the Turkish border in the summer of 2002. Most cases were reported between April and September. Increasing mean temperature, Normalized Difference Vegetation Index (NDVI), savannah-type land coverage or habitat fragmentation increased significantly the incidence of CCHF in the CCHF-affected areas. Similar to that observed in Turkey, we found that areas with warmer temperatures in the autumn prior to the case-reporting year had an increased probability of reporting zero CCHF cases.

**Conclusions:**

We identified environmental correlates of CCHF incidence in Bulgaria that may support the prospective implementation of public health interventions.

## Background

Crimean-Congo Haemorrhagic Fever (CCHF) is a zoonotic disease caused by a Nairovirus of the Bunyaviridae family that is usually transmitted by the bite of ixodid ticks, particularly of the *Hyalomma* spp. [1,2], or through contact with tissues, blood or other bodily fluids from infected people and animals [3,4].

*Hyalomma* ticks feed only once at each developmental stage. To serve as a CCHF vector, they must become infected at one stage, transmit the virus transtadially to the next stage or transovarially to the next generation, and then transmit the virus by bite to another vertebrate host [5]. Environmental changes can influence both the survival and reproduction of *Hyalomma* ticks, as well as trigger community outbreaks [6].

CCHF is one of the most feared viral haemorrhagic fevers because of its high fatality rate in human cases (up to 30%, depending on the transmission route) [1,7,8] and the potential to cause both community and nosocomial outbreaks [3]. The ratio of sub-clinical to clinical manifestations is approximately 5:1; thus, up to 80% of infections may be asymptomatic [5,9]. The seroprevalence of CCHF in people with a history of tick bite may be as high as 20% in endemic areas [10]. High-risk groups include farmers, livestock breeders, veterinarians, border army units, abattoir and healthcare workers [11]. Because of exposure to tick bites, outdoor recreational activities are also a recognized risk factor for CCHF in endemic areas [11].

CCHF is endemic in the Balkan area, including Bulgaria [1,12]. Most cases occur between April and September in the southeast and the south-central part of the country, with a limited number of cases involved per outbreak [1]. These cases follow by approximately one month the appearence of adult forms of *Hyalomma marginatum*, the main vector species of CCHF in Bulgaria [1]. *H. marginatum* is believed to be ubiquitous in Bulgaria (Christova I, personal communication). However, field data on its geographical distribution are not available.

The first documented outbreak in Bulgaria occurred during the agricultural collectivization in 1953 [1]. After the introduction of a vaccination programme of high-risk groups of the population in 1974 [12], cases decreased from 1,105 in 1953–1974 to 279 in 1975–1996 [8,13], to 196 in 1997–2008 [10]. Although the overall number of cases decreased over time, a major outbreak was observed in 2008, with a cluster of cases in the southwest part of Bulgaria, an area historically considered at low risk for CCHF outbreaks [13].

Poverty, human migrations, lack of vector control and inadequate management of infected patients among healthcare workers have been associated with an increased risk of acquiring CCHF [5,6,11,14]. Furthermore, neglect of agricultural lands, agricultural reforms resulting in landscape alterations and global warming may play a relevant role in CCHF emergence as well, although so far no empirical proof has been provided to support this latter claim [15,16].

In animals, including livestock and hares, CCHF is asymptomatic [12]. Domestic ruminants (e.g. cattle, sheep and goats) are considered to be short-lived reservoirs of the virus [1], and hares are important amplifying hosts, as they are commonly infested with immature stages of ticks [1,8]. The enzootic cycle of CCHF therefore depends on a complex network of ticks and host populations, and it is likely to be much more widespread than that revealed by the occurrence of clinical cases [17]. Generally speaking, the risk of CCHF infection in humans increases suddenly when the potential for enzootic transmission and/or the degree of exposure increase as well; thus, the investigation of those environmental factors that may influence the cycle of CCHF is relevant for outbreak preparedness and response [12].

In this study, we aimed at identifying those environmental determinants that may support the development of risk-based surveillance activities for CCHF in Bulgaria. Specifically, we investigated the association between the incidence of CCHF in humans and a selection of landscape and climatic factors that may be relevant to explain the occurrence of CCHF cases in Bulgaria.

## Methods

### Human data

A retrospective study was carried out to identify cases of CCHF that occurred in the resident population of Bulgaria between 1997 and 2009. Cases were collected from surveillance registry data and by contacting local authorities. Cases were laboratory confirmed by viral detection using virus cultivation AND/OR serology by ELISA and complement fixation assay.

Information on age, gender, month of disease onset, and district of residence of the cases was collected from clinical records. Resident population figures for each district of Bulgaria were obtained from the census data of the year 2006. Cases and respective underlying populations were subsequently allocated to each municipality of the districts they belonged to, by month and year of disease onset.

### Environmental data

Environmental variables used in the study had a potential impact on *Hyalomma* tick biology and therefore on the possible occurrence of human CCHF cases. Environmental data for the study area, i.e. Bulgaria with its 253 municipalities, were collected from several sources for the period 2001–2009.

Data on the monthly mean land surface ambient temperature (°C) and medium resolution NDVI, a measure of photosynthetic activity and large-scale vegetation distribution [18], were obtained from the NEO (NASA Earth Observations) datasets (http://neo.sci.gsfc.nasa.gov). These are layers derived from radiances measured by the NOAA-AVHRR orbiting satellites that provide well-calibrated datasets at a resolution of 0.1° already corrected for atmospheric and orbital disturbances. Both temperature and NDVI values are calculated by NEO as the monthly mean of daily maximum values. Municipality-averaged monthly values of temperature and NDVI were calculated for each month and year of the study period.

Landscape information was obtained from the 2004–2006 ESA Globcover Land Cover layer derived from an automatic and regionally-tuned classification of time series of MERIS-FR composites. This land cover classification is broken into 22 land cover classes which are defined according to the UN Land Cover Classification System (LCCS) [19]. For each municipality, the percent coverage of each land cover class was calculated. A landscape fragmentation index was also defined as the total number of different land cover classes (i.e. patch categories) that fell in the same municipality, normalized by the corresponding municipality area.

Data on cattle and sheep density (number of animals per Km^2^) were obtained from the FAO’s gridded livestock density datasets (http://www.fao.org/AG/againfo/resources/en/glw/GLW_dens.html). Animal densities are calculated by the FAO from official census and survey data accounting for the amount of land suitable for livestock production. Based on the statistical relationship with some environmental variables in similar agro-ecological zones, livestock densities are predicted to match national census totals for the year 2005 according to the FAOSTAT database and are provided at a resolution of 0.05° [20]. Cattle and sheep mean densities were calculated for each municipality.

All spatial data manipulations were performed using ArcMap in ArcGIS (ESRI Inc. Redlands, CA, USA).

### Statistical analysis

As the space-time distribution of CCHF cases was positively skewed with a high proportion of zeros, zero-inflated modelling was used to deal with these count data. Zero-inflated models account for “excess zeros” (i.e. zero counts greater than expected based on the non-zero counts) by assuming that the zeros arise from two different processes [21,22]. The first process occurs with probability pi and produces only zeros (structural zeros), whereas the second process occurs with probability (1 − pi) Pr(Ki = 0), where Pr(Ki = 0) is a Poisson or Negative Binomial (NB) probability function with the zero events occurring by chance (sampling zeros). The overall probability of zero counts is a combination of the probabilities of the two processes. Positive counts (i.e. greater than zero) are assumed to occur with probability (1 − pi) Pr(Ki = yi), where Pr(Ki = yi) is a Poisson or NB probability function for the positive counts [21,22]. In our study, the structural zero process accounted for those districts in which there were no CCHF cases at all during the entire study period (apparently “CCHF-free” areas). The sampling zero process accounted for those districts were the counts of CCHF cases were either zero or values greater than zero depending on the month and year in question during the entire study period.

As the overdispersion parameter α was significantly different from zero and the Vuong likelihood ratio test revealed that the zero-inflated model was preferred against the standard negative binomial model, a Zero-Inflated Negative Binomial (ZINB) model was chosen, as it accounted both for the excess zeros and for the extra heterogeneity in the outcome [21,22].

The probability of the structural zero process and the number of event counts in the sampling zero process may depend on different covariates [21,22]. This allowed us to explain the occurrence of CCHF cases differently: the covariates in the zero-inflated term of the ZINB model modelled the left tail of the case distribution and explained the zero counts in the “CCHF-free” areas, whereas those in the NB part of the ZINB model modelled the right tail and explained the positive counts in the “CCHF-affected” areas. A logit link was used for the excess zeros and a log link for the mean of the NB distribution.

In the NB part of the ZINB model (sampling zero process), the following covariates were included to explain the positive counts of CCHF cases: 1) monthly mean temperature; 2) monthly mean NVDI; 3) habitat fragmentation index (low, medium, and high, according to tertiles), 4) percent coverage of grasslands and areas with predominant scrub and herbaceous vegetation; 5) livestock density; 6) year of the study period (treated as linear splines [23,24]); and 7) human density (individuals per Km^2^).

In the logit part of the ZINB model (structural zero process), the mean temperature in October-November of the previous year and the year of the study period (treated as linear splines) were included to explain the excess of zero cases, as it has been shown that late autumn temperatures before the sudden drop in winter temperatures may differ significantly between areas with and without reported human cases of CCHF [18].

The underlying human population was included as exposure variable in the ZINB model. The model accounted for within-municipality correlation resulting from repeated measurements made over time on the same municipalities using robust variance estimators. Statistical analysis was performed using STATA 10 (StataCorp LP, Lakeway Drive, TX, USA), and the significance level was set to 0.05.

## Results

Between 1997 and 2009, 159 cases (38 women and 121 men) were identified (Table 1), including 42 fatal cases (case fatality rate: 26%). The average age (years) of cases was 52.80 ± 17.32 standard deviation (median: 54.50). Cases were mainly reported from April to September 

**Table 1:** Number of cases, mean annual incidence per 100,000 and respective 95% confidence intervals (CI) for CCHF in Bulgaria between 1997 and 2009 by district

	**N° cases**	**Incidence**	**95%CI**
BRG – Burgas	71	0.59	(0.47; 0.74)
GSO – Sofia city	1	0.02	(0.00; 0.18)
HKS – Haskovo	25	0.21	(0.14; 0.30)
LVC – Lovech	3	0.02	(0.01;0.05)
MKH – Mikhaylovgrad	0		
PLD – Plovdiv	25	0.14	(0.07; 0.17)
RZG – Razgrad	0		
SOF – larger Sofia area	12	0.04	(0.02; 0.07)
VAR – Varna	22	0.16	(0.11; 0.25)
West (GSO, SOF, PLD)	38	0.08	(0.05; 0.10)
East (BRG, HKS, VAR)	118	0.31	(0.26; 0.37)
Other areas (LVC, MKH, RZG)	3	0.01	(0.00; 0.03)
**Total**	**159**	**0.13**	**(0.11; 0.15)**

Cumulative incidence of CCHF was 2.07 per 100,000 population. The incidence per 100,000/year (1997–2009) was 0.13 (95% confidence interval [CI]: 0.11-0.15). There were large variations in CCHF incidence among the districts (Table 1). Values ranged from 0.02 (95% CI: 0.00-0.18) to 0.59 (95% CI: 0.47-0.74) per 100,000/year (1997–2009). The incidence did not vary substantially between 1997 and 2009: the mean incidence per 100,000/year was 0.13 (95% CI:0.10-0.17) during 1997–2000; 0.20 (95% CI: 0.16-0.25) during 2001–2004; and 0.08 (95% CI: 0.06-0.11) during 2005–2009.

**Figure 1:**
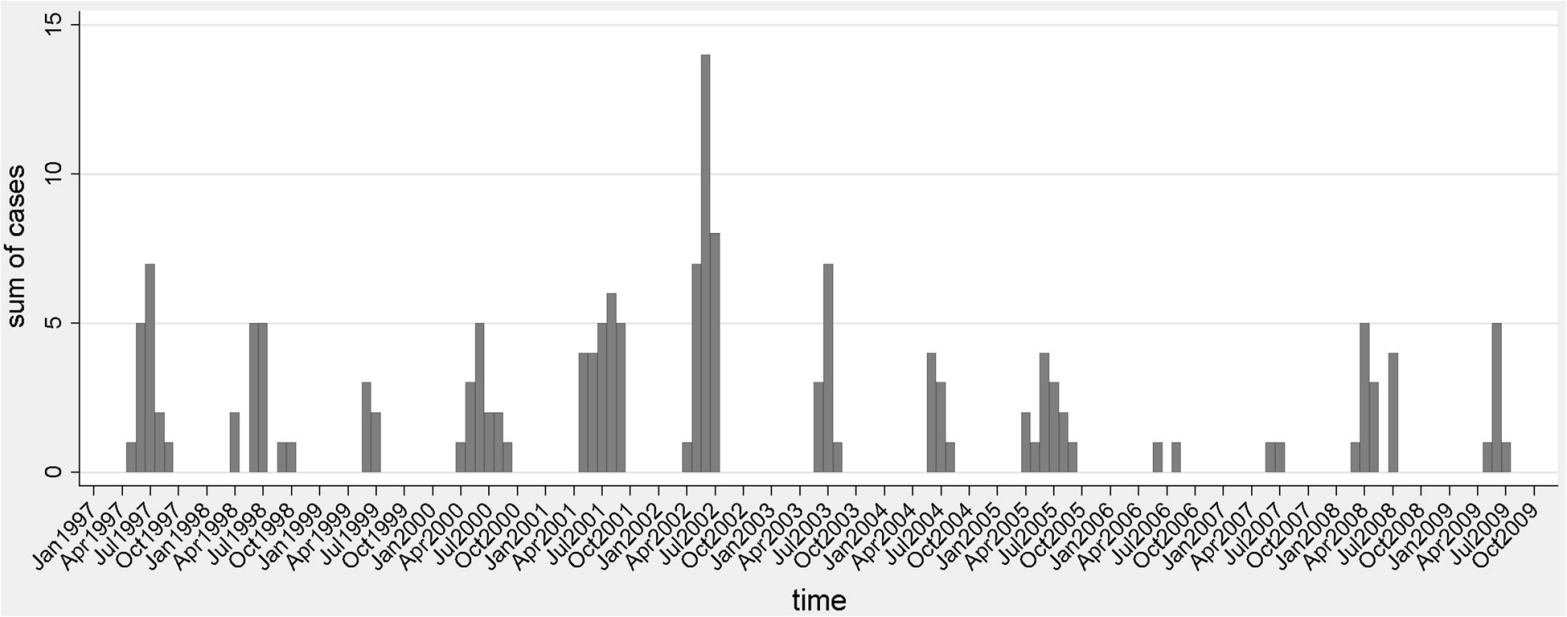
Distribution of CCHF cases by month and year of occurrence.

The temporal pattern of CCHF cases from 1997 to 2009 showed recurrent peaks in the summer, with a main peak in the summer of 2002 in the southeast and northeast districts of the country, particularly the districts of Stara Zagora, Burgas, Varna, and Haskovo, where 30 cases were reported within the same month of June (Figure 1). The first cases occurred in the districts of Stara Zagora and Burgas, close to the borders with Turkey. In 2008, another cluster of 12 cases was reported in the southwest part of the country, an area previously considered at low risk for CCHF. 

Results from the ZINB model are reported in Table 2. According to the model characteristics, the results are presented for its two constitutive parts separately: the first part (Table 2-A), based on the sampling zeros process, shows the pattern of associations between the explanatory variables and the incidence of CCHF in the “CCHF-affected” areas, while the second part (Table 2-B), based on the structural zero process, presents the association of the selected explanatory variable with the consistent presence of zero CCHF cases in the “CCHF-free” areas**.**

**Table 2:** Incidence rate ratios (IRR), Odds ratios (OR) and 95% confidence intervals (CI) from the zero inflated- negative binomial regression model

**A - Negative binomial regression for counts of CCHF cases**	IRR*	**95%CI**	**P-value**
**Mean temperature (°C)**	1.055	(1.048; 1.062)	< 0.001
**Mean NDVI**	1.018	(1.014; 1.022)	< 0.001
**Habitat fragmentation level**			
**low**	1		
**medium**	1.402	(1.120; 1.754)	0.003
**high**	1.558	(1.137; 2.137)	0.006
**Proportion of areas covered by grasslands, scrub and herbaceous vegetation**			
**low**	1		
**medium**	3.994	(1.812; 8.803)	0.001
**high**	4.260	(1.960; 9.260)	< 0.001
**Cattle density**	0.986	(0.966; 1.006)	0.160
**Sheep density**	1.001	(0.992; 1.010)	0.853
**Human density**	0.815	(0.521; 1.278)	0.374
**B - Logistic regression for excess of zero CCHF cases**	OR*	**95%CI**	**P-value**
**Mean temperature (°C) in October-November of the previous year**	1.129	(1.015; 1.257)	0.026

*Adjusted by calendar year.

In those districts where CCHF cases were observed, a unit increase in the mean temperature and NDVI values was significantly associated with an increase in the incidence rate by 5.5% (Incidence Rate Ratio [IRR]: 1.055), and by 1.8% (IRR: 1.018), respectively. Compared to areas with a lowly fragmented land structure, those with a high and medium level of fragmentation were significantly associated with an increase in CCHF incidence (IRR: 1.558; and IRR: 1.402, respectively). In the districts with a high and medium coverage of grassland, scrub and herbaceous vegetation, the incidence was 4.261 and 3.994 times the incidence of the reference group (i.e. the districts where this type of vegetation was less represented). There was no evidence of association between CCHF incidence and human density, cattle density, and sheep density.

The absence of CCHF cases in the “CCHF-free” areas was significantly associated with the mean temperature from October to November (Odds Ratio [OR]: 1.129), meaning that the higher the temperatures in October-November the higher the probability of reporting zero cases in the following year.

## Discussion

Bulgaria has been challenged by CCHF since the Second World War, becoming only the European country in which the disease is so far officially considered to be endemic, with recurrent epidemics particularly in the southeast part of the country. A similar epidemiological situation is increasingly being documented also in neighbouring countries, such as Albania, Kosovo, Russia, Turkey, Ukraine and Greece [25].

In this study, we described recent patterns in CCHF incidence in Bulgaria and investigated the extent to which the considered environmental factors were associated with the observed pattern of CCHF incidence in Bulgaria between 2001 and 2009, using a zero-inflated modelling approach.

### Explaining zero counts of CCHF cases

There were many areas with zero cases of CCHF during the entire study period. This occurs frequently with vector-borne diseases, as transmission to humans may depend on several biotic and abiotic factors as well as their interactions that may vary substantially over space and time. Moreover, it has been shown that CCHF does not occur in all the areas characterized by a suitable habitat for *H. marginatum,* but only in a narrow subset of them, as observed during the Turkish epidemic in 2003–2008 [18].

We observed that increasing mean temperature in late autumn (i.e. October-November) increased the likelihood of reporting zero counts of CCHF cases in the following year. A similar finding has previously been observed in Turkey, where an analysis of the environmental factors that differed between areas with and without CCHF cases showed that the areas with CCHF cases had colder mean temperatures in the late autumn and winter (November-February) compared to those without CCHF cases [18]. As immatures moult in the late autumn at that latitude, high temperatures after moulting would trigger their questing activity for hosts, whereas low temperatures would send them into quiescence. It has therefore been hypothesized that in areas with colder temperatures after moulting, more abundant overwintering tick populations would be synchronously activated in the spring of the following year, thereby increasing the potential for viral circulation [18].

### Explaining non-zero counts of CCHF cases

We found that the incidence of CCHF increased with increasing mean temperature in areas with CCHF cases. Temperature has previously been identified as the main factor driving the seasonal pattern and abundance of *H. marginatum*[26,27], as high temperatures, especially in the spring and summer, may accelerate *H. marginatum* cycle by switching on its interstadial development [28] and host questing activity [3,26,28]. Moreover, humans may adjust their recreational and subsistence outdoor activities according to the weather, increasing the chance of encountering and being bitten by these ticks [14,29].

The incidence of CCHF was higher in those areas characterized by a high proportion of grasslands, scrub and herbaceous vegetation (savannah-type environment), where in fact *Hyalomma* ticks usually find their ecological optima [2,30].

Livestock density was not significantly associated with CCHF incidence. In South Africa, the prevalence of antibody against CCHF virus has been found to be associated with the handling of sheep [1], suggesting that people with occupational exposure to livestock are particularly at-risk for CCHF. However, veterinarians and farm workers are usually vaccinated against CCHF in Bulgaria, and this may be the reason as to why this association was insignificant in our study.

Areas with a higher incidence of CCHF were those characterized by a highly fragmented habitat. A similar association has been found in previous studies [18,31,32], supporting the hypothesis that a fragmented land structure may increase the risk of acquiring CCHF by favouring viral circulation and amplification though frequent at-risk contacts between ticks, humans, livestock and wildlife [18]. Habitat fragmentation has also been identified as a critical factor for the distribution of Lyme disease [33], and recent modelling studies have suggested that increasing host biodiversity (species richness) may decrease the risk of acquiring Lyme disease through a dilution effect, whereas habitat fragmentation may increase this risk by reducing wildlife biodiversity [34]. However, it is difficult to conceptualize how the dilution effect and the impact of reduced biodiversity actually apply to CCHF, a disease characterized by a variety of different transmission and tick hosts. Possibly, the small fragments would increase appropriate habitat for ticks and their hosts, such as high densities of small mammals due to lower densities of predators and/or competitors, resulting in a critical rise in the rate of at-risk contacts.

We found a significant positive association between NDVI and CCHF incidence. A similar correlation has previously been found in Turkey [35], suggesting that the NDVI may proxy for tick seasonal activity. The NDVI reflects the amount and vigour of healthy green biomass during the seasonal cycle of vegetation as a surrogate of the seasonal fluctuations in weather and has long been recognized as one of the most important environmental variables influencing habitat suitability for ticks [32,36,37], as well as in regulating tick survival rates [27,38]. This is also partly due to the growth and dispersal rates of vertebrate host populations, particularly wild herbivores, which are strongly influenced by the quality and quantity of the available forage vegetation. Consistent with previous findings [39], the largest increase in NDVI occurred in the early growing season, i.e. the spring, but a relatively large NDVI was also found in the summer, which apparently accounted for the significant positive association with CCHF incidence in the “CCHF-affected” areas we found. As the effect of NDVI was still significant even after controlling for the land cover that best characterizes *H. marginatum* in the ZINB model, it is likely that the observed association with NDVI reflected the natural seasonal fluctuations of environmental exposure to abiotic factors (e.g. intensity and length to sunlight exposure) that can influence tick phenology and fitness, and consequently CCHF occurrence.

### Limitations

Infection rates of ticks and vertebrate hosts as well as frequency of at-risk contacts between humans and ticks are major determinants for CCHF infection in humans [40]. However, this kind of information and quantitative data on the abundance of ticks and vertebrate reservoirs (i.e. non-migratory terrestrial mammals, such as hares, small rodents, hedgehogs and resident bird species) were not available in our study. This may have limited our capacity to fully explain the observed pattern of CCHF in Bulgaria.

Another limitation is the use of administrative boundaries (i.e. districts and municipalities) as territorial units, as the lack of coordinates of cases did not allow for the use of other geographical resolutions. However, the use of such territorial units allowed for intra-unit variability in both biotic and abiotic factors, making it possible to generalize model predictions. This is because infection may have been acquired away from home, but possibly within the same municipality/district of residence location. Moreover, quality of data for probable sites of exposure (point locations) may vary according to differences in information gathering practice and interest of individual physicians, especially regarding asymptomatic or mild cases who have not been recognized as such, thereby precluding reliable use of any point locations as the basis for determining environmental correlates of CCHF incidence in Bulgaria.

## Conclusions

This study employed both GIS-derived information and classical statistical techniques to investigate CCHF patterns in Bulgaria and found that increasing mean temperature, NDVI, savannah-type land coverage and habitat fragmentation increased significantly the risk of CCHF in the “CCHF-affected” areas. Similar to that observed in Turkey, the temperature in the late autumn prior to the case-reporting year seemed to influence the probability of occurrence of zero CCHF cases in the “CCHF-free” areas. However, to further investigate the driving environmental factors behind CCHF patterns, data on tick abundance and infection rates, wildlife populations and societal factors are required.

There is urgent need to improve both passive and active surveillance activities for CCHF in Bulgaria and to evaluate vaccine coverage in high-risk areas. A holistic approach to risk assessment of CCHF under global climate change is also required to estimate the impact on, and the vulnerability of, human populations in relation to ticks and the prevalence of infection in vertebrate hosts under different environmental scenarios [41].

Despite the wealth of research documenting the widespread distribution, diversity of possible vectors and potential reservoirs for CCHF, our understanding of its epidemiology remains incomplete and at times we can only speculate on the importance of the different variables that can influence CCHF transmission cycle and stimulate outbreaks. In this regard, the factors underlying the spread of CCHF in Bulgaria we found, although they have not yet fully clarified, can make a substantial contribution towards fulfilling the information needs of public health policy-makers when challenging CCHF in varying eco-epidemiological dimensions.

## Competing interests

The authors’ declare that they have no competing interests.

## Authors’ contribution

FV, LB, LMG and GR conceived and designed the study, performed/interpreted the analysis, and prepared the manuscript for publication. IC and ET carried out the laboratory work, collected the data and contributed to the preparation of the manuscript. CK and LA supported the interpretation of results and contributed to the preparation of the manuscript. All authors have read and approved the final version of the manuscript.

## Key points

CCHF is endemic in Bulgaria where it causes either sporadic cases and community outbreaks.

A total of 159 CCHF cases (38 women and 121 men) were identified between 1997 and 2009 (incidence: 0.13 cases per 100,000 population/year; fatality rate: 26%).

Increasing temperature, NDVI, savannah-type land coverage, and habitat fragmentation increased the risk of CCHF in areas affected from the disease.

Areas with lower winter temperatures were associated with an increased probability of reporting zero CCHF.

## Pre-publication history

The pre-publication history for this paper can be accessed here:

http://www.biomedcentral.com/1471-2458/12/1116/prepub
